# Pharmacist-led medication reviews for geriatric residents in German long-term care facilities

**DOI:** 10.1186/s12877-019-1052-z

**Published:** 2019-02-11

**Authors:** Kerstin Bitter, Christina Pehe, Manfred Krüger, Gabriela Heuer, Regine Quinke, Ulrich Jaehde

**Affiliations:** 1 Department of Clinical Pharmacy, University of Bonn, An der Immenburg 4, 53121 Bonn, Germany; 2 AOK Rheinland/Hamburg Health Insurance, Kasernenstr. 61, 40213 Düsseldorf, Germany; 3 Linner Apotheke, Rheinbabenstraße 170, 47809 Krefeld, Germany; 4 Pharmacists’ Association North Rhine, Tersteegenstr. 12, 40474 Düsseldorf, Germany

**Keywords:** Drug-related problems, Long-term care facilities, Medication review, Medication safety, Nursing homes, Pharmacists

## Abstract

**Background:**

The benefit of medication reviews for long-term care (LTC) residents has been generally recognized throughout health care systems. Whereas many studies showed the impact of comprehensive medication reviews performed by specialized clinical pharmacists, little is known about the impact of medication reviews performed by community pharmacists. Involving them in the provision of medication reviews may help satisfy the increasing demand for ensuring medication safety.

**Methods:**

Community pharmacists supplying drugs to the LTC facilities performed a medication review for German LTC residents aged at least 65 years and taking five or more drugs per day based on the patients’ medication only. Documented potential drug-related problems (DRPs) and the implementation rate of pharmaceutical interventions were evaluated descriptively. To assess the quality of the medication reviews, we developed a corresponding reference system based on the analysis of two experienced clinical pharmacists.

**Results:**

Twelve pharmacies performed medication reviews for 94 LTC residents. Overall, the pharmacists documented 154 potential DRPs (mean 1.6 per patient, SD 1.5) of which the most common were drug-drug interactions (40%) followed by potentially inappropriate medication (PIM) (16%) and inappropriate dosages (14%). 33% of the pharmacists’ interventions to solve DRPs were successfully implemented, mostly dosage adjustments. The identification of potentially severe drug-drug interactions and PIM showed the highest agreement (88 and 73%) with the reference system.

**Conclusions:**

The medication review program of community pharmacists for LTC residents led to the identification of relevant DRPs. The reference system assessing the quality of the service can contribute to its transparency and reveals the potential for its improvement. The community pharmacists’ knowledge of the LTC residents and their relation to the prescribers is crucial for providing successful medication reviews.

## Background

A medication review is a structured evaluation of a patient‘s medicines by detecting drug-related problems (DRPs) and recommending interventions [1]. Whereas medication reviews as part of the Medicines Use Review Service or the Medication Therapy Management Program are already implemented in the United Kingdom and the United States, in Germany they have not yet become an established health care service. Statutory health insurances (SHI), insuring about 90% of the German population, do not yet pay medication review providers apart from the scope of studies or pilot projects so that pharmacists still demand remuneration as a condition to provide medication reviews. Contracts are negotiated with the providers’ federal or state associations ensuring ‘sufficient, appropriate and economical’ services pursuant to the German social legislation [2]. Selective contracting, considered one of the most successful tools of the managed care techniques in the US, is not as effective for German insurers since collective contracts are still mandatory [3]. Regardless of the differences in health care systems and the diverse remuneration concepts, it is important for insurers, whether private or statutory, to know about the quality of the services they pay for. Whereas many studies showed the impact of pharmacist-led medication reviews on reducing potential DRPs and improving appropriate polymedication [4–8], little is known about the quality of the service provided under routine conditions. The proof of a well-performed service could be the basis for remuneration in general or a condition for entering a contract to get a higher level of remuneration compared to non-contracted providers.

It is anticipated that geriatric patients profit most from medication reviews as they are generally at risk of suffering from adverse drug events [9]. In German long-term care (LTC) facilities for instance, the incidence of adverse drug events was found to be 7.9 per 100 resident-months of which about 60% were judged preventable [10]. This corresponds to studies in LTC facilities in the United States and Canada observing 9.8 adverse drug events per 100 resident-months, 42% considered preventable [9].

In Germany, LTC facilities can enter supply contracts with pharmacies. Thus, a medication review service performed by pharmacists supplying drugs to LTC facilities might be favourable due to the pharmacists’ knowledge of the residents’ current medication and their relationship with the prescribers, advantages that were also seen for consultant pharmacists conducting medication reviews in LTC facilities in the US [11]. The aim of this study was to evaluate the results and the quality of medication reviews provided by community pharmacists for residents of LTC facilities and to derive recommendations for the implementation of this service into routine healthcare practice. The study might be of interest for health care institutions and researchers not only in Germany but also in other countries developing and establishing medication reviews in LTC settings.

## Methods

Between 2014 and 2016 we conducted a cross-sectional study on potential DRPs among German LTC residents in North Rhine-Westphalia, the most populous federal state of Germany. One of the leading statutory health insurances (SHIs) in this area, the ‘Allgemeine Ortskrankenkasse (AOK) Rheinland/Hamburg’, contracted with the Pharmacists’ Association North Rhine and the University of Bonn to conduct this study. Ethics approval was obtained.

More than 900 community pharmacies of the area were informed about the study by a circular letter and could apply for participation without any further conditions than supplying drugs to a LTC facility. Of about 200 applicants, 17 pharmacies were drawn randomly. Residents of the pharmacies’ cooperating LTC facilities were invited to participate if they were members of the aforementioned SHI, aged at least 65 years and taking five or more prescribed drugs per day (inclusion criteria). Informed consent was obligatory for participation. As this was a feasibility study, a formal sample size calculation was not performed.

### Intervention

The pharmacists got a half-day training focussing on medication reviews in older patients and auxiliary materials to perform an extended version of a ‘simple medication review’, according to the definition of the Pharmaceutical Care Network Europe [1]. Originally, this type of medication review is based solely on the medication history, enabling the pharmacist to detect drug-drug interactions, double medication and contraindication due to age or gender. In addition to the medication history, pharmacists in this model had access to prescription data obtained from the SHI and dosing schedules from the LTC facility. Therefore, further DRPs were detectable. Table 1 shows all detectable DRP categories and the corresponding criteria for evaluation.

**Table 1:** Content of the Medication Review for LTC patients

DRP category	Criteria for evaluation
Drug-drug interactions (DDI)	• DDI categorized potentially severe • DDI categorized less severe but considered relevant, e.g. for the therapy’s effectiveness or due to additive adverse effects
Double medication	• Double administration of the same active drugs or drug class if not considered plausible
Inappropriate dosage interval	• Dosage interval not corresponding to the SPC if considered relevant, e.g. for the prevention of adverse drug effects
Inappropriate administration time	• Administration time not corresponding to the SPC if considered relevant, e.g. for the therapy’s effectiveness
Potentially inappropriate medication (PIM)	• Drug from the PRISCUS list [13] taking dose-dependence into account, and • drug effect mainly systemic, and • alternative drug available
Inappropriate dosage form	• Product that is divided but not divisible according to the SPC
Inappropriate dosage	• Dosage/strength not recommended for any indication or not age-adjusted according to the SPC and/or PRISCUS list
Inappropriate duration of use	• Duration of use not recommended according to the SPC
Indication to start a drug treatment	• Medication indicating the need for another drug, e.g. for the prevention of adverse drug effects
Contraindication comorbidity	• Drug not generally recommended due to a comorbidity that is obvious from the medication
Others	• e.g. use of a recalled product

**Fig. 1:**
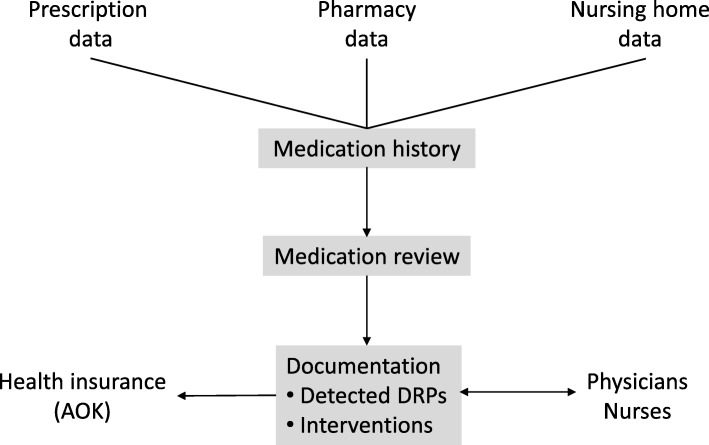
Process of the intervention (AOK ‘Allgemeine Ortskrankenkasse’, German statutory health insurance; DRP drug-related problem)

Each step of the intervention and the exchange of information between the involved parties is shown in Fig. 1.

The first step of the medication review was to document a current medication history for every patient, including over-the-counter drugs purchased in the participating pharmacy which are usually recorded for registered patients in the pharmacy’s software. Subsequently, the pharmacists conducted the medication review documenting all identified DRPs and interventions. Physicians or nurses were informed on pharmaceutical recommendations by using specific forms. In order to reveal if the recommendations had been implemented, pharmacists were asked to document changes of the patients’ medication eight to twelve weeks after the intervention.

Consequently, the final report comprised the patients’ age and gender, the original and updated medication history, identified DRPs and interventions. The report was sent to the SHI and remunerated once per patient. The SHI pseudonymized and forwarded the pharmacists’ reports to the University of Bonn where these were used for evaluation.

### Evaluation

The evaluation focussed on the number and type of documented DRPs and pharmaceutical interventions. The implementation rate of interventions was examined for patients with follow-up medication data. However, this data does not enable to evaluate the implementation of monitoring recommendations. Therefore, the implementation of such interventions was unknown.

To assess the quality of the medication reviews, every medication history was forwarded to two experienced clinical pharmacists who reviewed the medication independently without insight into the community pharmacists’ results. After consensus finding in case of inconsistencies between the experts’ reviews, the result was called ‘Reference DRPs’. The Reference DRPs served as a standard of comparison and were used for assessing the ‘agreement rate’ defined as the number of DRPs documented by the pharmacists related to the number of Reference DRPs. At the end of the study, an acceptance analysis was conducted by evaluating the pharmacists’ responses to a questionnaire about the feasibility of the service using a 4-point Likert scale.

Statistical data analysis was conducted using Microsoft Excel™ (Microsoft Corporation, Redmond, USA). The number of drugs was evaluated at the level of active drugs using the WHO Anatomical and Therapeutic Classification [12].

## Results

### Study population

**Fig. 2:**
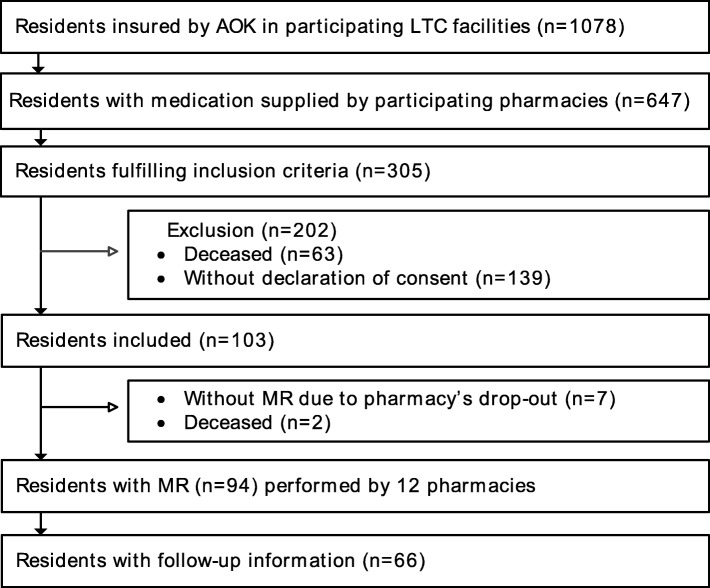
Patient recruitment (AOK ‘Allgemeine Ortskrankenkasse’, German statutory health insurance; LTC long-term care; MR medication review)

One thousand seventy-eight SHI members living in one of 20 participating LTC facilities were screened. 305 of them fulfilled the inclusion criteria and were informed about the study (see Fig. 2).

34% (103) signed the informed consent and for 94 patients a medication review was performed by one of 12 community pharmacies. The pharmacists documented follow-up medication data for 66 of the patients. Reasons for non-conducted medication reviews (*n* = 9) were the residents’ decease after inclusion (*n* = 2) or study withdrawal of their pharmacies (*n* = 7) due to closure, extensive workload or termination of their collaboration with the LTC facility.

The residents’ mean age was 84 years, 66% were female. On average, they took 13 different drugs per day (SD 3), self-medication included. 35% took at least one regularly scheduled drug considered as potentially inappropriate medication in older persons (PIM) according to the PRISCUS list, the German equivalent to the Beers Criteria [13, 14].

### Intervention

The pharmacists performed seven medication reviews on average, ranging from three to 17. They documented 154 DRPs (mean 1.6 DRPs per patient, SD 1.5) of which the most common were drug-drug interactions (40%), followed by PIM (16%) and inappropriate dosages (14%). The mean number of documented DRPs differed between the pharmacies and ranged from 0.5 to 3.5 per patient and pharmacy. Additionally, the pharmacists revealed 69 documentation errors in the LTC facility concerning the medication, which was e.g. not up-to-date (27.5%) or lacking information about the correct administration time (27.5%).

Compared to the pharmacists’ documented DRPs, the number of reference DRPs was higher (mean 2.5, SD 1.9), ranging from 1.0 to 3.8 per patient and pharmacy.

Documented interventions (*n* = 131) mainly concerned monitoring issues (25%), followed by recommendations to change a dosage regimen (19%) or to discontinue a certain drug therapy (18%).

33% of the pharmacists’ interventions to solve DRPs were successfully implemented, mostly dosage adjustments. The implementation rate varied among pharmacies in a range of 11–88% while the implementation of every fifth intervention was unknown. 81% of the interventions were recommendations to the physicians. Interventions accepted by the physicians (*n* = 18) were principally cessations of a certain drug therapy (*n* = 6) and dosage adjustments (*n* = 5), mainly concerning drugs acting on the nervous system (WHO ATC group N), leading to a reduction of PIM of 18%. Recommendations concerning drug substitutions were mostly rejected.

### Quality

**Fig. 3:**
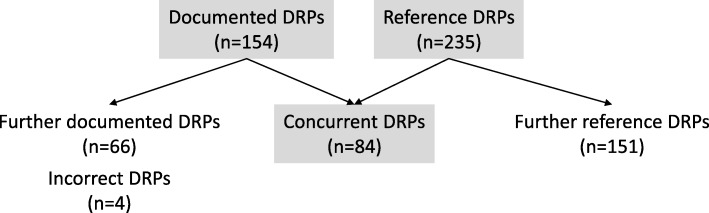
Primary endpoint drug-related problems **(**DRP drug-related problem)

Out of 235 reference DRPs, 84 (36%) were concurrent with the pharmacists’ documented DRPs (see Fig. 3).

**Fig. 4:**
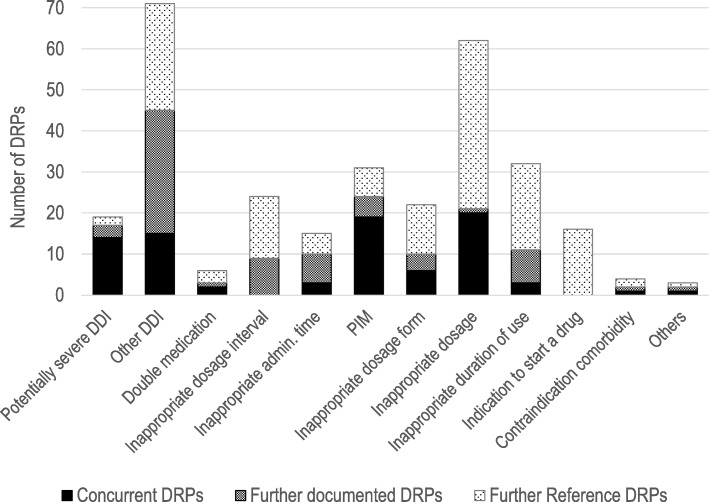
Type and number of documented, reference and concurrent DRPs (DDI drug-drug interaction, DRP drug-related problem, PIM potentially inappropriate medication in older adults according to the PRISCUS list [13])

The DRP category with the highest agreement rate (88%) in our study concerned potentially serious drug-drug interactions (see Fig. 4).

The second-best agreement rate (73%) was observed related to the identification of PIM. Only two pharmacies did not detect existing PIM. DRPs concerning dosages were only detected by half of the participating pharmacists documenting a third of the reference DRPs. No agreement was observed in DRPs concerning dosage intervals. Pharmacists documented deviations from the Summaries of Product Characteristics that expert reviewers considered of low clinical relevance. Regarding the agreement rate, we found a high variability between the participating pharmacists (15–100%).

Of all documented DRPs, the expert reviewers considered only four (3%) to be incorrect. All other documented DRPs that differed from the reference DRPs (*n* = 66) were not rated to be incorrect but of minor clinical relevance.

### Acceptance analysis

Only one of the twelve pharmacists did not participate in the acceptance analysis resulting in a return rate of exploitable questionnaires of 92%. The evaluation of the preparation including the prior training and provided auxiliary material was mostly positive (93%). Although limited time resources were claimed by 64% of the pharmacists, 82% considered the service rather successful in their own setting. Moreover, 100% of the pharmacists derived great satisfaction stating that the service did not only improve their pharmacy’s image but also enriched their pharmaceutical work.

## Discussion

The geriatric population of this study reflects a representative sample of LTC residents in Germany with regard to their demographic characteristics [15]. Due to the narrow spectrum of detectable DRP categories by means of a simple medication review, the number of DRPs and thereby interventions in our study was low compared to others [16–19].

Furthermore, the implementation rate was relatively low [9, 18, 20]. On the one hand, this is surprising because the study design made use of existing inter-professional relationships and the pharmacists self-evaluated their collaboration with the GPs rather positive (64%) than negative (36%). On the other hand, the lack of information within the scope of a simple medication review might not be beneficial to promote inter-professional collaboration as it is difficult for pharmacists to judge the clinical relevance of potential DRPs without further patient information or laboratory data. Accordingly, the provision of increasing clinical information showed an improvement of the medication reviews’ quality [21]. Focussing on clinically relevant DRPs may promote the collaboration with the GPs since recommendations with impact on patient outcomes support the pharmacists’ trustworthiness [22–25]. For instance, it might have been annoying for GPs to get recommendations of alternative drugs that were not suitable for the patient’s indication or of a therapy monitoring that the GP already conducted. This might be a reason for the frequent rejection of recommendations concerning drug substitutions in this study.

Only one pharmacy in our study got more than half of their interventions implemented. This pharmacy showed also a good agreement rate (78%) and the lowest proportion (18%) of DRPs considered of minor clinical relevance by the expert reviewers. The low error rate of documented DRPs concurs with previous study results (1 and 4%) evaluating the correctness of either the community pharmacists’ identified DRPs or their recommendations [26, 27].

Regarding the agreement rate with the reference system, it has to be considered that the identified reference DRPs depended on the medication reported by the pharmacists. Because of their knowledge about the patients and prescribers, community pharmacists might have had relevant additional information which they did not document so that reference DRPs and documented DRPs are not completely comparable. For instance, based on the pharmacists’ previous experiences with the prescribers, they might have ignored DRPs expecting that the GPs would not implement their recommendation anyway. However, the overall agreement rate was similar to the results of other studies evaluating medication reviews conducted by community pharmacists [21, 27, 28]. A higher agreement rate can probably be achieved by more extensive prior training [26]. The potential influence of the training may also explain the good results concerning the identification of PIM since it was one of the main topics during the pharmacists’ training in this study. Greissing et al. found a lower agreement rate between DRPs identified by untrained German community pharmacists and predefined DRPs in a fictitious patient example [29]. In that study, 97% of 143 participating pharmacists did not identify PIM. The pharmacists’ reliable detection of potentially serious drug-drug interactions can be explained by the fact that all pharmacists used a software to detect drug-drug interactions. The pharmacists documented a much lower number of DRPs concerning inappropriate dosages compared to the reference DRPs. Since indications were unknown, dosage recommendations could only be general or referred to age or gender. Too general dosage recommendations might have hindered pharmacists to intervene. The same goes for DRPs concerning indications to start a drug as these are not generally detectable within the scope of a simple medication review. Examples of reference DRPs of this category were the indication for a laxative in combination with an opioid or the indication for a proton pump inhibitor during long-term therapy with non-steroidal anti-inflammatory drugs.

The high variability among the pharmacies concerning the agreement rate can be due to the different workload in the individual pharmacies. Since patients were invited for participation all at once, pharmacies with a higher number of study participants might have performed the medication reviews under time pressure. Supporting this assumption, two of three pharmacies with the most medication reviews stated that they would not have had the capacity to perform more medication reviews than they did. Another factor that could have influenced the agreement rate is professional competence. Pharmacists were asked for self-assessment of their expertise in performing medication reviews. Only one pharmacy stated to lack required knowledge to perform medication reviews. Actually, this pharmacy showed a low agreement rate (16%). Since this pharmacy conducted the highest number of medication reviews (18% of all), its results contributed decisively to the overall results whereas the pharmacy with the best agreement rate (100%) conducted only 6% of all medication reviews. However, the small sample size did not allow for further examination of influence factors of the medication reviews’ results and inter-professional collaboration.

## Conclusions

The study showed that pharmacists detect many DRPs in LTC residents even by conducting medication reviews based solely on the medication history. However, there was still room for improvement concerning the quality of the medication reviews performed by community pharmacists. The results are likely to be representative for other German community pharmacists with the restriction that study pharmacists could have differed from the average in terms of motivation.

The low implementation rate of the pharmacists’ interventions suggests that the medication review service could become more effective if the pharmacists’ collaboration with the physicians improved. A higher implementation rate may be achieved if pharmacists get extended access to further information enabling them to give more recommendations relevant to patient outcomes and thus gain the GPs’ appreciation. Feedback on the individual performance, e.g. by an external quality assessment like in this study, could be helpful in the process of developing the required skills.

## Abbreviations

AOK: ‘Allgemeine Ortskrankenkasse’ (German statutory health insurance)
DDI: Drug-drug interactions
DRP: Drug-related problem
LTC: Long-term care facility
PIM: Potentially inappropriate medication in older persons
SHI: Statutory health insurance
